# In vivo formation of *N*-acyl-fumonisin B_1_

**DOI:** 10.1007/s12550-014-0211-5

**Published:** 2014-10-19

**Authors:** Henning Harrer, Hans Ulrich Humpf, Kenneth A. Voss

**Affiliations:** 1 Institute of Food Chemistry, Westfälische Wilhelms-Universität, Münster, Germany; 2 Toxicology & Mycotoxin Research Unit, USDA Agricultural Research Service, 950 College Station Road, Athens, GA 30605-2720 USA; 3 Russell Research Center, USDA, Agricultural Research Service, Athens, GA USA

**Keywords:** Fumonisin, Metabolism, Mycotoxin, Biodistribution, *N*-acyl-fumonisins

## Abstract

Fumonisins are fungal toxins found in corn and in corn-based foods. Fumonisin B_1_ (FB_1_) is the most common and is toxic to animals, causes cancer in rodents, and is a suspected risk factor for cancer and birth defects in humans. The hydrolyzed form of FB_1_ (HFB_1_) also occurs in foods and is metabolized by rats to compounds collectively known as *N*-acyl-HFB_1_ (also known as *N*-acyl-AP_1_). *N*-acyl-HFB_1_ is structurally similar to ceramides, metabolites which have important structural and signaling functions in cells. FB_1_ is *N*-acylated in vitro to ceramide-like metabolites which, like FB_1_, are cytotoxic. However, metabolism of FB_1_ and inhibition of ceramide synthase by its metabolites in vivo has not been demonstrated. Male rats were dosed ip with 0.5, 1, or 2 mg/kg body weight FB_1_ on five consecutive days and the liver and kidney thereafter processed for chemical analysis. *N*-acyl derivatives of fumonisin B_1_ were identified for the first time in these principal target organs of FB_1_ in rats, at levels up to 0.4 nmol/g tissue using mass spectrometry. The *N*-acyl chain length of the metabolites varied in a tissue-dependent manner with C_16_ derivatives predominating in the kidney and C_24_ derivatives being prevalent in the liver. The toxicological significance of *N*-acyl-fumonisins is not known and warrants investigation.

**Electronic supplementary material:**

The online version of this article (doi:10.1007/s12550-014-0211-5) contains supplementary material, which is available to authorized users.

## Introduction

Fumonisins are secondary metabolites of fungi, mainly *Fusarium verticillioides* and *Fusarium proliferatum*. They were described for the first time by Gelderblom et al. (1988) who isolated and characterized fumonisin B_1_ (FB_1_) by means of a biosassay based on the promotion of carcinogenesis in rat liver. Further studies demonstrated that FB_1_, the most prevalent congener, is hepato- and nephrotoxic to rodents (Voss et al. 1989; Voss et al. 2001). In addition, several species-specific syndromes are caused by FB_1_. These include neural tube defects in the LM/Bc mouse (Voss et al. 2009; Gelineau-van Waes et al. 2009; Gelineau-van Waes et al. 2005; Gelineau-van Waes et al. 2012), equine leukoencephalomalacia (Marasas et al. 1988), and pulmonary edema in pigs (Harrison et al. 1990). Fumonisins are structurally similar to the sphingoid bases sphinganine and sphingosine, and Wang et al. (1991) found that FB_1_ inhibits ceramide synthase (CerS), a critical enzyme in the de novo biosynthesis of ceramide and complex sphingolipids, and disrupts sphingolipid metabolism. These and subsequent studies have established disrupted sphingolipid metabolism as the mode of action of fumonisins (Bulder et al. 2012).

In a previous study, Humpf et al. (1998) found that hydrolyzed FB_1_ (HFB_1_), an alkaline hydrolysis product of FB_1_ that is present in some foods, is a substrate for CerS in rat liver microsomes (Humpf et al. 1998). Specifically, HFB_1_ substitutes for sphinganine or sphingosine so that CerS catalyzes the acylation of HFB_1_ at the primary amino group with fatty acids of various chain lengths to form ceramide analogs known as *N*-acyl-HFB_1_ (NAHFB_1_). HFB_1_ was also metabolized in vivo: NAHFB_1_ of various fatty acyl chain lengths were found in the liver and kidney of rats exposed to HFB_1_ (Seiferlein et al. 2007). Harrer et al. (2013), using an optimized mass spectrometry method, more recently demonstrated the in vitro formation of *N*-acyl-FB_1_ (NAFB_1_) in human cell lines which had been transfected for the overexpression of CerS. We now report the in vivo formation of NAFB_1_ in rats exposed to FB_1_.

## Materials and methods

### Animals and experimental design

The study protocol was approved by the Institutional Animal Care and Use Committee, Richard B. Russell Agricultural Research Center, Athens, GA. Male Sprague–Dawley rats (Harlan Industries, Indianapolis, IN, USA), 7 weeks of age at receipt, were individually housed in stainless steel, wire mesh cages in an environmentally controlled room having a 12-h light/dark cycle. Food (2019 Global Rodent Diet, Teklad, Madison, WI, USA) and fresh tap water were provided ad libitum. After a 1 week acclimation period, the animals were randomly assigned to five groups (*n* = 2/group) having mean weights of 215 to 216 g (weight range of all animals, 211 to 220 g). For five consecutive days, they were observed, weighed, and given an intraperitoneal injection (ip) of 0 (vehicle), 0.5, 1.0, or 2.0 mg/kg body weight FB_1_ (provided by R. Eppley, US FDA), 1.0 mg/kg body weight HFB_1_ (positive control) (Voss et al. 2009), or vehicle (0.9 % physiological saline). These doses corresponded to 0.69, 1.38, and 2.77 μmol FB_1_/kg body weight and 2.47 μmol/kg body weight HFB_1_ per day. In order to compare the effects of FB_1_ and HFB_1_, dosages will be expressed as molar concentrations and, on this basis, the high dose of FB_1_ is roughly equivalent to that of the positive control dose of HFB_1_. Dosing solutions were sterile filtered and administered at a volume rate of 10 ml/kg body weight. The animals were fasted overnight prior to administration of the final dose. The rats were euthanized (CO_2_ inhalation and exsanguination) 60 to 90 min following the final dose administration and examined by necropsy. The kidney and liver specimens were fixed in 10 % neutral buffered formalin, processed, and microscopically evaluated without knowledge of the animal’s identity or treatment group. Three representative liver and kidney specimens (100 mg each) were also collected, immediately frozen, and stored (−80 °C) until processed, at which time the tissues were thawed and homogenized in distilled/deionized water (1 ml water/100 mg tissue). The homogenates were lyophilized before they were shipped by overnight courier for the analysis of fumonisin metabolites and sphingolipids.

### Chemicals and reagents

Reverse phase columns were from Phenomenex (Aschaffenburg, Germany) and Varian (Darmstadt, Germany). All chemicals and solvents were analytical grade and purchased from Sigma-Aldrich (Steinheim, Germany). Sphingolipid standards are from Avanti Polar Lipids (Alabaster, USA). FB_1_, HFB_1_, NAFB_1_, NAHFB_1_, and the isotope-labeled standard FB_1_-d_6_ were isolated and synthesized with methods described earlier (Lukacs et al. 1996; Hübner et al. 2012; Harrer et al. 2013).

### Quantification of NAFB_1_, NAHFB_1_, and sphingolipids

Lyophilized liver and kidney tissues (100 mg wet weight equivalents) were processed for the quantification of FB_1_, HFB_1_, NAFB_1_, NAHFB_1_, and sphingolipids. Preparation of the tissue samples was performed according to a previously published method (Harrer et al. 2013). For the quantification of FB_1_ and HFB_1_, a d_6_-labeled derivative of FB_1_ (FB_1_-d_6_) was used as the internal standard. Synthetic derivatives with a heptadecanoic fatty acid (C_17:0_) residue were used as internal standards for the quantification of *N*-acyl-fumonisins and ceramides (*N*-C_17:0_-FB_1_, *N*-C_17:0_-HFB_1,_ and *N*-C_17:0_-ceramide). The sphingoid bases and their phosphate derivatives were quantified using synthetic derivatives with a C_17_-acyl-backbone: sphingosine (C_17:1_), sphingosine-1-phosphate (C_17:1_), sphinganine (C_17:0_), sphinganine-1-phosphate (C_17:0_) as internal standards. The standards were dissolved in methanol:chloroform (2:1, *v*/*v*) and then mixed with the tissue samples. The samples spiked with internal standard were kept at room temperature for 30 min before being processed further. The internal standard-spiked samples were first extracted with ethyl acetate, isopropanol, and water (60:30:10, *v*/*v*/*v*) and a second time using methanol, chloroform, and water (60:30:10, *v*/*v*/*v*). The extracts were combined, the solvents removed by evaporation, and the dried samples stored at−80 °C till analyzed.

For the analysis, the samples were reconstituted in a mixture of water, methanol, and tetrahydrofurane (60:24:16, *v*/*v*/*v*). The NAFB_1_ and NAHFB_1_ derivatives having the following fatty acyl chain lengths were quantified by HPLC-MS/MS: C_16:0_, C_18:0_, C_20:0_, C_22:0_, C_24:0_, C_26:0_. Furthermore, we also quantified the unsaturated derivatives C_24:1_ and C_26:1_. The results are given as the sum of all *N*-acyl derivatives of FB_1_ or HFB_1_. Quantitative results for each individual *N*-acyl derivative are given in Table S1 of the Supplementary Material.

## Results and discussion

### Histological examination of rat liver and kidney

Mild to moderate apoptotic, mitotic, and other effects consistent with fumonisin exposure (Bulder et al. 2012; Voss et al. 2001) were found in all rats given FB_1_. Dose-related differences in severity were not obvious, likely as a consequence of the small sample size of *n* = 2/group and dosing regimen (levels and exposure route). In agreement with reports that HFB_1_ is less toxic than FB_1_ in rodents (Gelderblom et al. 1993; Collins et al. 2006; Howard et al. 2002; Voss et al. 2009), the microscopic appearance of the kidneys and liver from rats given 1.0 mg/kg body weight HFB_1_ per day could not be differentiated from that of tissues from the vehicle control group.

### Tissue FB_1_ and HFB_1_ concentrations

**Fig. 1:**
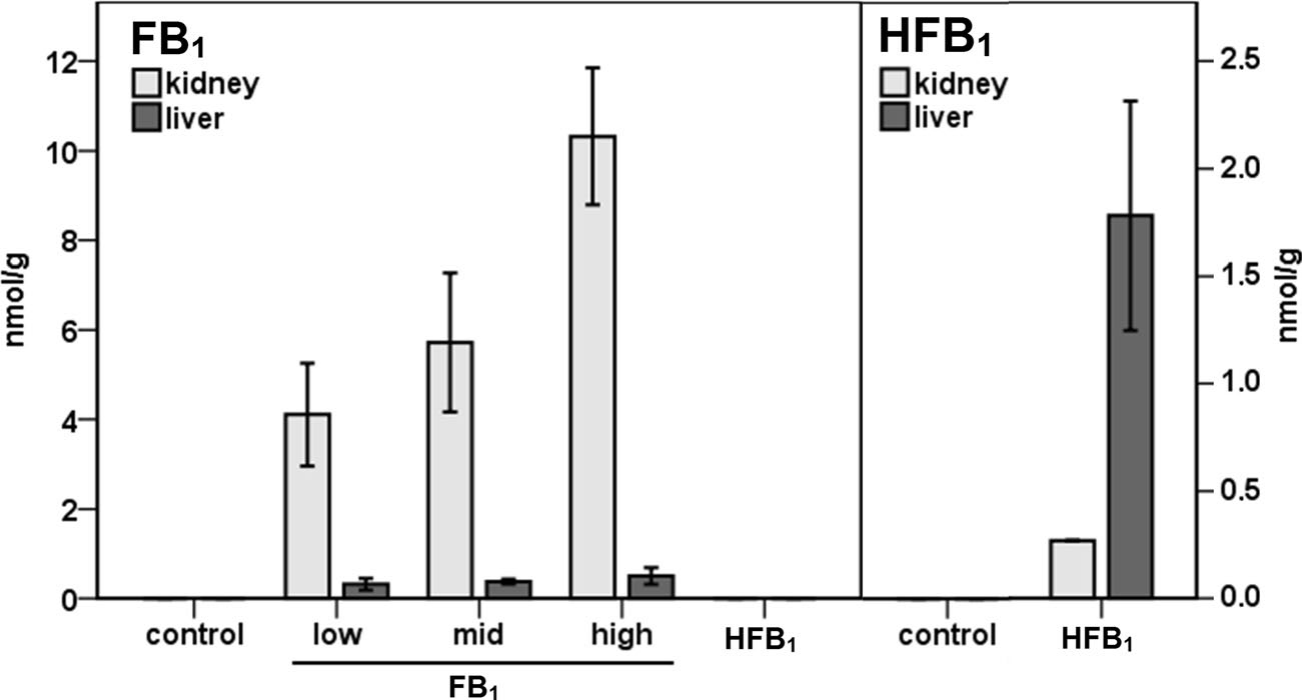
The molar concentrations of FB_1_ and HFB_1_ in the kidney and liver of rats. The animals were treated by intraperitoneal injection of FB_1_ or HFB_1_ for 5 days with doses of 0.69, 1.38, and 2.77 μmol FB_1_/kg body weight per day and 2.47 μmol/kg body weight HFB_1_ per day. The dose of HFB_1_ is the molar equivalent to the high dose group of FB_1_. FB_1_ and HFB_1_ in the tissues were quantified by HPLC-ESI-MS/MS. Values are means ± S.D. (*n* = 2). Note the difference in scale, *y*-axis. FB_1_ or HFB_1_ were not detected in tissues of vehicle-treated (control) rats

In order to evaluate the metabolism of fumonisins, the concentrations of FB_1_, HFB_1_, and their metabolites were determined in the kidney and liver. No fumonisins or metabolites were detected in the animals given the vehicle (see Fig. 1). In the FB_1_-exposed groups, a dose-dependent increase in FB_1_ concentrations was found in the kidneys that ranged from 4 to up to 10 nmol/g tissue. The average concentrations of FB_1_ in the liver were 12- to 20-fold lower (see Fig. 1). This pattern of preferential accumulation of FB_1_ in the kidney is consistent with previously published data (Riley and Voss 2006) showing that the FB_1_ concentration (ng/g wet weight) in the kidney of rats fed with fumonisin-contaminated diet was approximately tenfold higher than that in the liver. Our findings are also consistent with those of a single-dose toxicokinetic study of FB_1_ (Martinez-Larranaga et al. 1999) in which area under the concentration–time curve (AUC)tissue/AUCplasma ratios of 29.9 and 2.03 were determined for the kidney and liver, respectively.

A different pattern was found for HFB_1_. Its molar concentration in the liver was about sixfold higher than the concentration in the kidney (1.78 nmol/g in the liver and 0.27 nmol/g in the kidney, see Fig. 1).

### In vivo formation of NAFB_1_ and NAHFB_1_

**Fig. 2:**
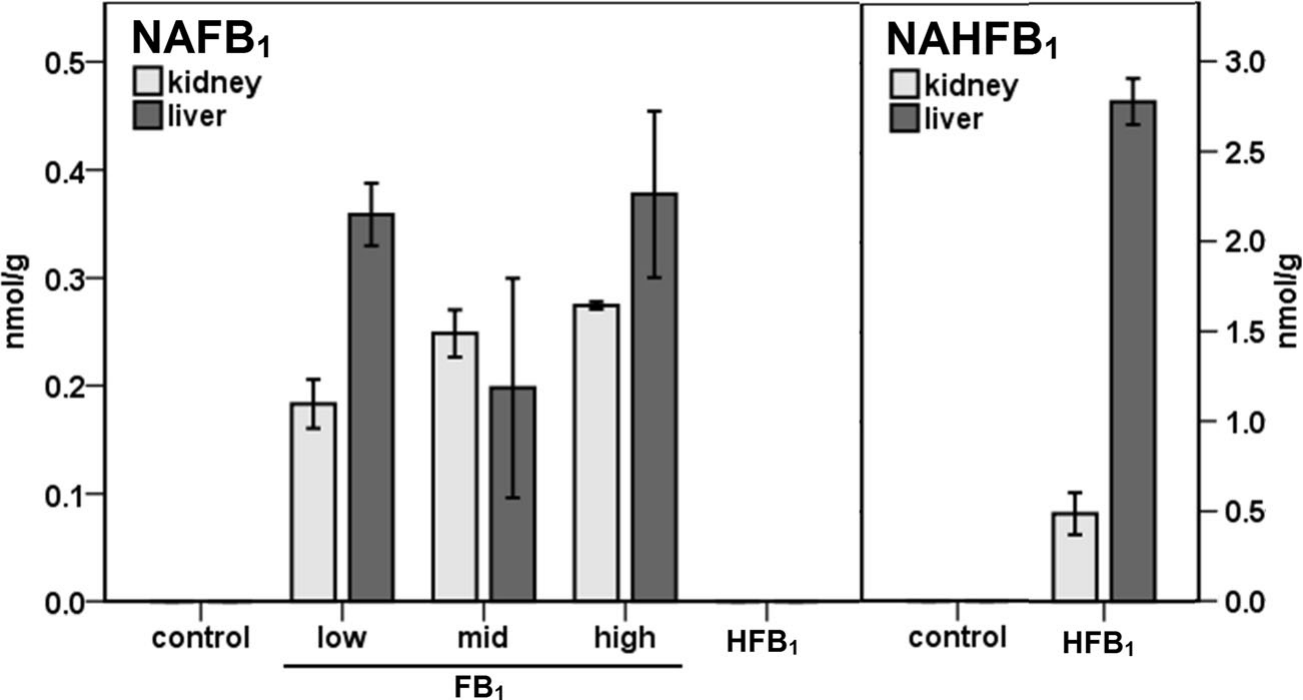
The concentrations of NAFB_1_ and NAHFB_1_ in the kidney and liver of rats (expressed as the sum of all *N*-acyl derivatives). Animals were treated by intraperitoneal injection of FB_1_ or HFB_1_ for 5 days with doses of 0.69, 1.38, and 2.77 μmol FB_1_/kg body weight per day or 2.47 μmol/kg body weight HFB_1_ per day. The dose of HFB_1_ is the molar equivalent to the high dose group of FB_1_. NAFB_1_ and NAHFB_1_ were analyzed by HPLC-ESI-MS/MS. Values are means ± S.D. (*n* = 2). Note the difference in scale, *y*-axis

*N*-acyl derivatives of fumonisins having various acyl chain lengths were found in animals treated with FB_1_ or HFB_1_, demonstrating for the first time the in vivo formation of *N*-acyl-FB_1_ in fumonisin’s two main target organs in rats (see Fig. 2). No metabolites have been observed in the vehicle-treated (control) animals.

The NAFB_1_ levels (expressed as the sum of all *N*-acyl derivatives) varied only slightly and ranged from 0.18 to 0.38 nmol/g. The amount of HFB_1_ metabolites in the liver was sixfold higher than that in the kidneys: the organs’ respective NAHFB_1_ concentrations were 2.8 and 0.5 nmol/g tissue (see Fig. 2). Very low amounts of NAHFB_1_ (<0.1 nmol/g tissue) were also detected in the tissues of animals given FB_1._ Their source was not determined although it can be speculated that they resulted from absorption and metabolism of HFB_1_ that had been formed from hydrolysis of FB_1_ by the intestinal microbiota (Shephard et al. 1994 and 1995). However, the possibility that enzymatic hydrolysis of small amounts of absorbed FB_1_ provided a pool of HFB_1_ in the tissues cannot be discounted.

### Total FB_1_ and HFB_1_ species in the kidney and liver

**Fig. 3:**
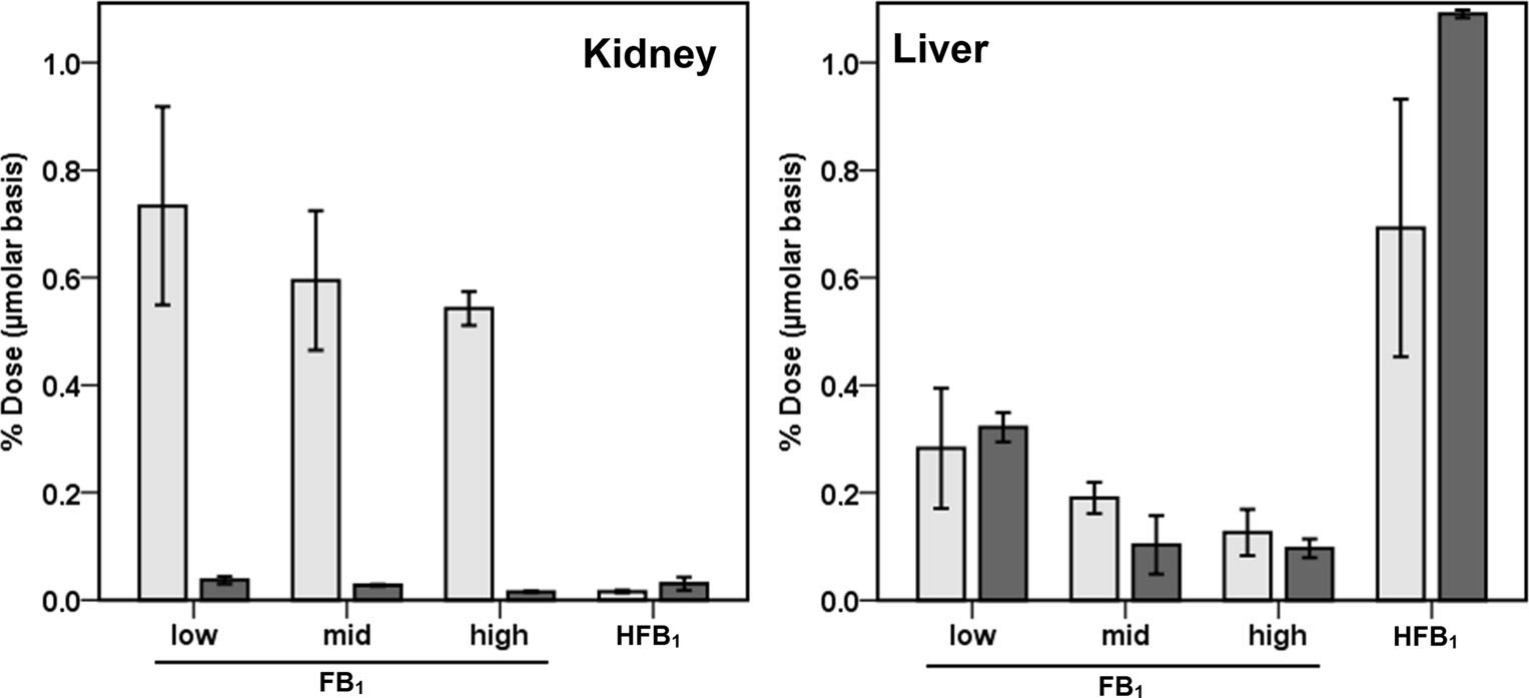
The relative accumulation of FB_1_ and HFB_1_ (*gray bars*) and their *N*-acyl metabolites (*black*) in the kidney and the liver after 5 days of exposure to the parent mycotoxin. The dose groups were 0.69, 1.38, and 2.77 μmol FB_1_/kg body weight per day and 2.47 μmol/kg body weight HFB_1_ per day. The dose of HFB_1_ is a (approximate) molar equivalent to the high dose group of FB_1._ Values are means ± S.D. (*n* = 2)

The tissue burdens of FB_1_, HFB_1_, and *N*-acyl metabolites in the kidney and liver after 5 days of exposure represented only a small fraction of the administered FB_1_ or HFB_1_. Differences in the accumulation in these target organs were apparent (see Fig. 3). Only 0.1 to 0.3 % of the total dose (TD) was recovered from the liver as unmetabolized FB_1_ while the amount found in the kidney was slightly higher, ranging from about 0.5 to slightly less than 0.8 % of TD. Compared to the high dose of FB_1_, a relatively high amount of unmetabolized HFB_1_, 0.7 % TD, accumulated in the liver and a relatively lower amount, <0.1 % TD of HFB_1_, in the kidney.

*N*-acyl-FB_1_ species in the liver ranged from 0.1 (low- and high-dose groups) to about 0.3 % TD (low-dose group) on a μmolar basis. Although low, these amounts represented 35 to 60 % of the total FB_1_ species in the liver (see Fig. 3). Only insignificant amounts of the FB_1_ species (<0.04 % TD and <10 % of FB_1_ species in the kidney) found in the kidneys were *N*-acyl metabolites. In contrast, *N*-acyl-HFB_1_ species in the liver accounted for 1.1 % TD, but, as was the case for FB_1_, this amount made up about 60 % of the total HFB_1_ species recovered in the liver. Similar to FB_1_ and its metabolites, the total amount of HFB_1_ species in the kidney was negligible and represented only 0.03 % TD. However, in contrast to the low contribution of metabolites to total renal FB_1_ species, 65 % of the HFB_1_ found in the kidney consisted of *N*-acyl metabolites.

The high dose of FB_1_ (2.77 μmol/kg body weight) and the HFB_1_ dose (2.47 μmol/kg body weight) were approximately equal, and, when comparing the results from these treatment groups, it was apparent that the kidney accumulated FB_1_ much more readily than HFB_1_. The results also suggest that the kidney has only a limited capacity to metabolize both mycotoxins as less than 0.5 nmol/g of FB_1_, or HFB_1_ metabolites were found. The male rat kidney is recognized as being extremely sensitive to the apoptotic and other effects of FB_1_ (Howard et al. 2001; Dragan et al. 2001). Nephrotoxicity has therefore served as a sentinel of exposure for applied studies (Burns et al. 2008; Voss et al. 2011) and has also provided a benchmark dose for risk assessment (Bulder et al. 2012).

### Tissue specificity of *N*-acylation of fumonisins

Different isoforms of CerS exist in the tissues of mammalian organisms. All are tissue specific, catalyze the *N*-acylation of sphinganine, and are specific for coenzyme A fatty acid cosubstrates having fatty acid residues that vary in length from C_14_ to C_26_ (Pewzner-Jung et al. 2006). We therefore analyzed the individual levels of ceramides and NAFB_1_ and NAHFB_1_ metabolites of different *N*-acyl chain lengths in the kidney and the liver of FB_1_- or HFB_1_-exposed rats.

**Fig. 4:**
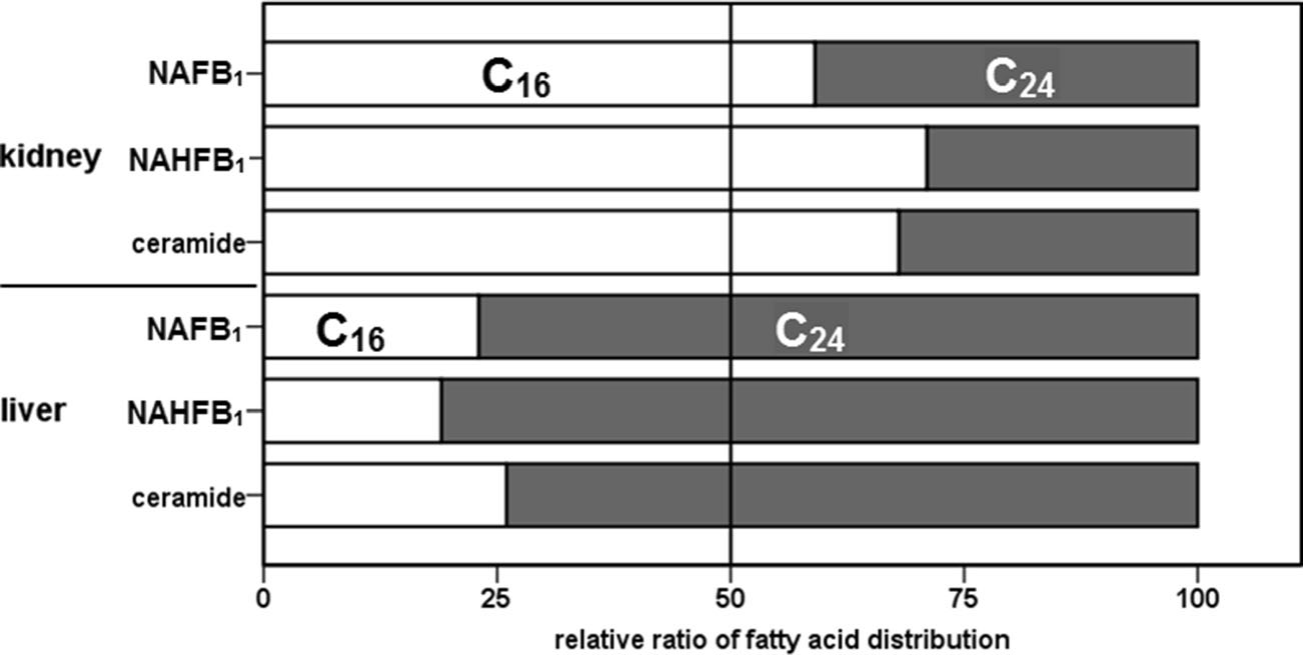
The distribution of the fatty acid chain lengths of *N*-acyl-FB_1_, *N*-acyl-HFB_1_, and ceramide in the kidney and liver of rats. Values are means (*n* = 2) and normalized to the sum of C_16_ and C_24_ derivatives found in the tissues (quantitative results for each individual *N*-acyl derivative can be found in Table S1 of the Supplementary Material)

The amounts of ceramide species found in the two tissues were different with C_16_ derivatives being more predominant in the kidney. In contrast, CerS isoforms in the liver generated mainly C_24_-ceramides (all quantitative results for each individual *N*-acyl derivative can be found in Table S1 the Supplementary Material). It is of interest that the ratio of *N*-C_16_- to *N*-C_24_-fumonisin derivatives in the kidney and liver was consistent with that of the ceramides found in each tissue (see Fig. 4). It can therefore be concluded that NAFB_1_ and NAHFB_1_ found in the liver and kidney were most likely products of reactions catalyzed by the CerS isoforms specific to those tissues. The gene expression of the CerS isoforms and the concordant ceramide pattern is well understood for the renal and hepatic tissue of mice. From the data of Laviad et al. (2008), it is seen that in the kidney of mice, the predominant ceramide species are the N-C_16_ derivatives and in the liver the N-C_24_ derivatives. Our data indicate that the ceramide pattern is consistent among mouse and rat tissues. However, there is no established knowledge about the chain length distribution and the gene expression of CerS isoforms in rats. Therefore, it could be interesting to verify these observations in further studies.

### Sphingolipids

**Fig. 5:**
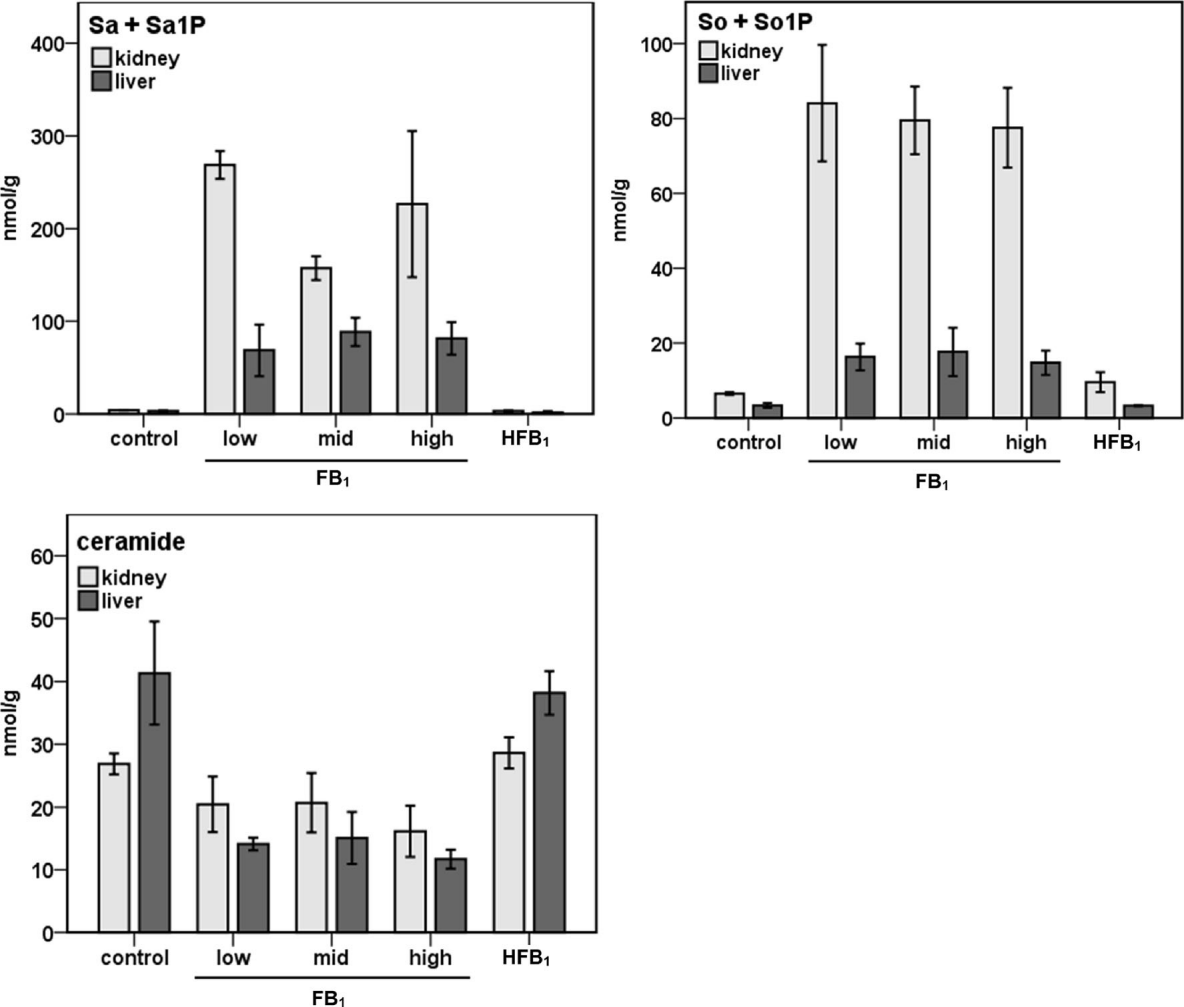
The levels of total sphinganine (*Sa*) plus sphinganine-1-phosphate (*Sa1P*), total sphingosine (*So*) plus sphingosine-1-phospate (*So1P*), and ceramide in rat kidney and liver. Animals were treated by intraperitoneal injection with doses of 0.69, 1.38, and 2.77 μmol FB_1_/kg body weight per day and 2.47 μmol/kg body weight HFB_1_ per day. The dose of HFB_1_ is the approximate molar equivalent of the high dose of FB_1_. The tissue samples were extracted, and all analytes were determined by HPLC-ESI-MS/MS. Values are means ± S.D. (*n* = 2)

Inhibition of CerS by FB_1_ is the key event in fumonisins’ mode of action (Dragan et al., 2001, Bulder et al., 2012). Inhibition disrupts overall sphingolipid metabolism, resulting in increased concentrations of sphinganine, sphingosine, and their 1-phosphates and decreased levels of ceramide and complex sphingolipids in tissues in vivo (Voss et al. 2009) and in cultured cells (Wang et al. 1991). The levels of several sphingolipids were therefore analyzed in the samples, and, as expected (Riley and Voss 2006), the rats responded to FB_1_ with increased amounts of total sphingosine (sphingosine plus sphingosine-1-phosphate), total sphinganine (sphinganine plus sphinganine-1-phosphate) (see Fig. 5), and the individual sphingoid bases and 1-phosphate metabolites in the tissues (see Table 1). In contrast, treatment with HFB_1_ did not cause any significant changes in the levels of the sphingolipids compared to the vehicle-treated controls (see Fig. 5 and Table 1). The FB_1_ modified sphingolipid profiles in the liver and kidney differently. The two sphingoid bases and their 1-phosphates were increased somewhat in the liver and much more extensively in the kidney whereas tissue ceramide concentrations were decreased in both tissues, with levels in the liver tending to be slightly lower (see Fig. 5 and Table 1). A limited number of in vitro studies have shown that NAHFB_1_ metabolites of HFB_1_ alter sphingolipid concentrations in various cell lines, including those overexpressing CerS isoforms (Seefelder et al. 2003; Harrer et al. 2013), and also were cytotoxic to IHKE human proximal tubule-derived cells (Seefelder et al. 2003) and other mammalian cell lines (Harrer et al. 2013). Whether or not NAFB_1_ and NAHFB_1_ metabolites contribute to toxicity in vivo is not known and requires further investigation. That HFB_1_ treatment in this study did not significantly alter tissue sphingolipid concentrations, is consistent with reports that it exerts a significantly lesser effect on tissue sphingolipid profiles, and is less toxic to rats and mice than FB_1_ (Collins et al. 2006; Howard et al. 2002; Voss et al. 2009).

**Table 1:** The levels of sphinganine (Sa), sphingosine (So), sphinganine-1-phosphate (Sa1P), and sphingosine-1-phospate (So1P) and the Sa/So ratio in rat kidney and liver. Animals were treated by intraperitoneal injection with doses of 0.69, 1.38, and 2.77 μmol FB_1_/kg body weight per day and 2.47 μmol/kg body weight HFB_1_ per day. The dose of HFB_1_ is the approximate molar equivalent of the high dose of FB_1_. The tissue samples were extracted, and all analytes were determined by HPLC-ESI-MS/MS. Values are means ± S.D. (*n* = 2)

Tissue	Substance	Dose	Sa nmol/g	So nmol/g	Sa/So ratio	Sa1P nmol/g	So1P nmol/g
Kidney	Control		2 ± 0.7	6 ± 0.4	0.4 ± 0.1	1.6 ± 0.7	1.0 ± 0.1
Kidney	FB_1_	Low	252 ± 15.6	63 ± 11.5	4.1 ± 1.0	16.7 ± 0.5	21.4 ± 4.1
Kidney	FB_1_	Mid	146 ± 11.3	59 ± 8.3	2.5 ± 0.2	11.3 ± 1.5	20.8 ± 0.7
Kidney	FB_1_	High	207 ± 79.9	56 ± 6.2	3.8 ± 1.8	19.9 ± 1.0	21.6 ± 4.5
Kidney	HFB_1_	High	3 ± 0.7	9 ± 2.8	0.3 ± 0.0	0.6 ± 0.4	0.4 ± 0.1
Liver	Control		3 ± 0.3	3 ± 0.6	1.0 ± 0.1	0.2 ± 0.0	0.3 ± 0.0
Liver	FB_1_	Low	68 ± 27.6	16 ± 3.3	4.2 ± 0.9	0.2 ± 0.2	0.4 ± 0.2
Liver	FB_1_	Mid	88 ± 15.1	17 ± 6.4	5.5 ± 1.2	0.4 ± 0.1	0.8 ± 0.0
Liver	FB_1_	High	81 ± 17.1	14 ± 2.9	5.9 ± 0.0	0.9 ± 0.5	1.0 ± 0.3
Liver	HFB_1_	High	2 ± 1	3 ± 0.2	0.5 ± 0.4	0.1 ± 0.1	0.2 ± 0.0

### Dosing route and relevance

Given the previous absence of evidence for in vivo FB_1_ metabolism (other than conversion to HFB_1_ by gut microflora) together with its low absorption after oral exposure (<5 % of dose) (reviewed by Bulder et al. 2012) and low conversion rate to NAFB_1_ in vitro (Harrer et al. 2013), multiple high ip doses were used in this “proof of concept” study. The extent to which the NAFB_1_ species are formed and accumulate in tissues after dietary FB_1_ exposure in animals or humans is not known, but is almost certain to be manyfold lower than that found in this study. It should further be recognized that the high doses of FB_1_ or HFB_1_ possibly compromised tissue metabolic or transport systems, thereby influencing metabolite production and retention. This possibility is especially relevant for the kidney of rats given FB_1_ because of the mild to moderate nephrotoxicity induced in these animals. Additional investigations following oral exposure to physiologically relevant doses are therefore needed to evaluate the role, if any, of NAFB_1_ in FB_1_ toxicity.

## Conclusions

This experiment has to our knowledge revealed for the first time the metabolic conversion of FB_1_ to a series of NAFB_1_ species in vivo. Consistent with earlier reports, much more FB_1_ was recovered from the kidneys than from the liver. Renal FB_1_ was however almost exclusively unmetabolized whereas approximately half of the FB_1_ in the liver consisted of *N*-acyl species, suggesting that metabolic turnover might occur more readily in the liver. Furthermore, the acylation pattern, that is, the chain lengths of the fatty acid moieties of the metabolites, recovered from the two organs differed, likely as a result of different isoforms of ceramide synthase that predominate in the two tissues. Metabolism of FB_1_ differed in some respects from that of HFB_1_. It is noteworthy that, in contrast to FB_1_, over half of the HFB_1_ species found in the kidney were metabolites, an observation suggesting the possibility that metabolism contributes to the relatively lower nephrotoxicity of HFB_1_. Sphingolipid metabolism was markedly disrupted by FB_1_ while tissues sphinganine, sphingosine, sphingoid base 1-phosphate, and ceramide concentrations in the tissues of rats dosed with HFB_1_ were similar to those in vehicle-treated rats. The contribution of fumonisin metabolites to toxicity is not known and warrants further study.

## Electronic supplementary material

Below is the link to the electronic supplementary material.

ESM 1 (DOC 175 kb)

## Abbreviations

FB_1_: Fumonisin B_1_
CerS: Ceramide synthase
ESI-MS/MS: Electrospray ionization-tandem mass spectrometry
HFB_1_: Hydrolyzed fumonisin B_1_
NAFB_1_: *N*-acyl-FB_1_
NAHFB_1_: *N*-acyl-HFB_1_
SL: Sphingolipid
TCA: Propane-1,2,3-tricarboxylic acid
