# Supplementary material for: In vivo formation of N-acyl-fumonisin B1
Source: Mycotoxin Res. 2014 Oct 19;31(1):33–40. doi: 10.1007/s12550-014-0211-5 (PMC4298654; doi:10.1007/s12550-014-0211-5)
Supplement: Supplementary file 1 — (DOC 175 kb) [file 12550_2014_211_MOESM1_ESM.doc]

## SUPPLEMENTARY MATERIAL

**In vivo formation of *N*-acyl-fumonisin B_1_**

Henning Harrer^1^, Hans Ulrich Humpf^1*^ and Kenneth A. Voss^2*^

^1^Institute of Food Chemistry, Westfälische Wilhelms-Universität, Münster, Germany

^2^USDA, Agricultural Research Service, Russell Research Center, Athens, GA, USA

***Correspondence:**

Dr. Kenneth A. Voss, USDA Agricultural Research Service, Toxicology & Mycotoxin Research Unit, 950 College Station Road, Athens, GA 30605, USA

E-Mail: Ken.Voss@ars.usda.gov

Fax: +1-706-546-3116

Professor Dr. Hans-Ulrich Humpf, Institute of Food Chemistry, Westfälische Wilhelms-Universität, Münster, Germany

E-Mail: humpf@uni-muenster.de

Fax: +49-251-83-33396

**Table S1** Quantitative results for each individual *N*‑acyl-derivative in the liver and kidney samples (each sample was analyzed twice, a and b represent the results for each individual analysis).

| treatment | | analysis | | | | | | | | | | | | | |
| --- | --- | --- | --- | --- | --- | --- | --- | --- | --- | --- | --- | --- | --- | --- | --- |
| compound | dose mg/kg BW | tissue | analyte | a | b | Mean |  | S.D. |  | analyte | a | b | mean |  | S.D. |
| FB_1_ | 0,5 | Kidney | HFB_1_C_16:0_ | 0,0 | 0,2 | 0,1 | ± | 0,11 |  | FB_1_C_16:0_ | 7,4 | 9,1 | 8,2 | ± | 0,82 |
| FB_1_ | 1 | Kidney | HFB_1_C_16:0_ | 0,2 | 0,1 | 0,2 | ± | 0,06 |  | FB_1_C_16:0_ | 14,4 | 12,6 | 13,5 | ± | 0,93 |
| FB_1_ | 2 | Kidney | HFB_1_C_16:0_ | 0,3 | 0,5 | 0,4 | ± | 0,12 |  | FB_1_C_16:0_ | 12,1 | 13,8 | 13,0 | ± | 0,82 |
| HFB_1_ | 1 | Kidney | HFB_1_C_16:0_ | 22,7 | 37,3 | 30,0 | ± | 7,30 |  | FB_1_C_16:0_ | 0,0 | 0,0 | 0,0 | ± | 0,00 |
| control | 0 | Kidney | HFB_1_C_16:0_ | 0,0 | 0,0 | 0,1 | ± | 0,06 |  | FB_1_C_16:0_ | 0,0 | 0,0 | 0,0 | ± | 0,00 |
| FB_1_ | 0,5 | Liver | HFB_1_C_16:0_ | 0,3 | 0,4 | 0,3 | ± | 0,05 |  | FB_1_C_16:0_ | 6,8 | 5,1 | 6,0 | ± | 0,87 |
| FB_1_ | 1 | Liver | HFB_1_C_16:0_ | 0,0 | 0,4 | 0,2 | ± | 0,22 |  | FB_1_C_16:0_ | 3,9 | 6,4 | 5,2 | ± | 1,29 |
| FB_1_ | 2 | Liver | HFB_1_C_16:0_ | 0,0 | 0,3 | 0,1 | ± | 0,15 |  | FB_1_C_16:0_ | 8,4 | 5,9 | 7,1 | ± | 1,22 |
| HFB_1_ | 1 | Liver | HFB_1_C_16:0_ | 34,9 | 44,3 | 39,6 | ± | 4,72 |  | FB_1_C_16:0_ | 0,0 | 0,0 | 0,0 | ± | 0,00 |
| control | 0 | Liver | HFB_1_C_16:0_ | 0,0 | 0,0 | 0,1 | ± | 0,01 |  | FB_1_C_16:0_ | 0,0 | 0,0 | 0,0 | ± | 0,00 |
| FB_1_ | 0,5 | Kidney | HFB_1_C_18:0_ | 0,1 | 0,2 | 0,2 | ± | 0,03 |  | FB_1_C_18:0_ | 2,5 | 2,9 | 2,7 | ± | 0,20 |
| FB_1_ | 1 | Kidney | HFB_1_C_18:0_ | 0,1 | 0,0 | 0,1 | ± | 0,06 |  | FB_1_C_18:0_ | 3,8 | 3,1 | 3,4 | ± | 0,33 |
| FB_1_ | 2 | Kidney | HFB_1_C_18:0_ | 0,0 | 0,1 | 0,1 | ± | 0,06 |  | FB_1_C_18:0_ | 4,0 | 2,8 | 3,4 | ± | 0,58 |
| HFB_1_ | 1 | Kidney | HFB_1_C_18:0_ | 2,6 | 1,8 | 2,2 | ± | 0,40 |  | FB_1_C_18:0_ | 0,0 | 0,0 | 2,1 | ± | 0,44 |
| control | 0 | Kidney | HFB_1_C_18:0_ | 0,0 | 0,0 | 0,0 | ± | 0,00 |  | FB_1_C_18:0_ | 0,0 | 0,0 | 1,6 | ± | 0,34 |
| FB_1_ | 0,5 | Liver | HFB_1_C_18:0_ | 0,3 | 0,1 | 0,2 | ± | 0,08 |  | FB_1_C_18:0_ | 3,1 | 2,4 | 2,8 | ± | 0,33 |
| FB_1_ | 1 | Liver | HFB_1_C_18:0_ | 0,0 | 0,0 | 0,0 | ± | 0,00 |  | FB_1_C_18:0_ | 2,0 | 3,0 | 2,5 | ± | 0,49 |
| FB_1_ | 2 | Liver | HFB_1_C_18:0_ | 0,0 | 0,0 | 0,0 | ± | 0,00 |  | FB_1_C_18:0_ | 3,0 | 3,9 | 3,5 | ± | 0,47 |
| HFB_1_ | 1 | Liver | HFB_1_C_18:0_ | 1,5 | 2,0 | 1,8 | ± | 0,27 |  | FB_1_C_18:0_ | 0,0 | 0,0 | 2,1 | ± | 0,32 |
| control | 0 | Liver | HFB_1_C_18:0_ | 0,0 | 0,0 | 0,0 | ± | 0,00 |  | FB_1_C_18:0_ | 0,0 | 0,0 | 2,2 | ± | 0,59 |
| FB_1_ | 0,5 | Kidney | HFB_1_C_20:0_ | 0,0 | 0,0 | 0,0 | ± | 0,00 |  | FB_1_C_20:0_ | 0,2 | 0,5 | 0,4 | ± | 0,12 |
| FB_1_ | 1 | Kidney | HFB_1_C_20:0_ | 0,0 | 0,1 | 0,1 | ± | 0,06 |  | FB_1_C_20:0_ | 0,2 | 0,0 | 0,1 | ± | 0,12 |
| FB_1_ | 2 | Kidney | HFB_1_C_20:0_ | 0,0 | 0,0 | 0,0 | ± | 0,00 |  | FB_1_C_20:0_ | 0,0 | 0,6 | 0,3 | ± | 0,30 |
| HFB_1_ | 1 | Kidney | HFB_1_C_20:0_ | 3,1 | 0,6 | 1,8 | ± | 1,23 |  | FB_1_C_20:0_ | 0,0 | 0,0 | 0,0 | ± | 0,00 |
| control | 0 | Kidney | HFB_1_C_20:0_ | 0,0 | 0,0 | 0,0 | ± | 0,00 |  | FB_1_C_20:0_ | 0,0 | 0,0 | 0,0 | ± | 0,00 |
| FB_1_ | 0,5 | Liver | HFB_1_C_20:0_ | 0,0 | 0,2 | 0,1 | ± | 0,12 |  | FB_1_C_20:0_ | 0,0 | 0,0 | 0,0 | ± | 0,00 |
| FB_1_ | 1 | Liver | HFB_1_C_20:0_ | 0,2 | 0,1 | 0,2 | ± | 0,00 |  | FB_1_C_20:0_ | 0,0 | 0,0 | 0,0 | ± | 0,00 |
| FB_1_ | 2 | Liver | HFB_1_C_20:0_ | 0,0 | 0,0 | 0,0 | ± | 0,00 |  | FB_1_C_20:0_ | 0,0 | 0,0 | 0,0 | ± | 0,00 |
| HFB_1_ | 1 | Liver | HFB_1_C_20:0_ | 8,9 | 13,1 | 11,0 | ± | 2,13 |  | FB_1_C_20:0_ | 0,0 | 0,0 | 0,0 | ± | 0,00 |
| control | 0 | Liver | HFB_1_C_20:0_ | 0,0 | 0,0 | 0,0 | ± | 0,00 |  | FB_1_C_20:0_ | 0,0 | 0,0 | 0,0 | ± | 0,00 |
| FB_1_ | 0,5 | Kidney | HFB_1_C_22:0_ | 0,0 | 0,0 | 0,0 | ± | 0,00 |  | FB_1_C_22:0_ | 0,7 | 0,7 | 0,7 | ± | 0,03 |
| FB_1_ | 1 | Kidney | HFB_1_C_22:0_ | 0,0 | 0,0 | 0,0 | ± | 0,00 |  | FB_1_C_22:0_ | 0,9 | 0,6 | 0,7 | ± | 0,15 |
| FB_1_ | 2 | Kidney | HFB_1_C_22:0_ | 0,0 | 0,0 | 0,0 | ± | 0,00 |  | FB_1_C_22:0_ | 0,9 | 1,4 | 1,2 | ± | 0,24 |
| HFB_1_ | 1 | Kidney | HFB_1_C_22:0_ | 1,5 | 4,3 | 2,9 | ± | 1,39 |  | FB_1_C_22:0_ | 0,0 | 0,0 | 0,0 | ± | 0,00 |
| control | 0 | Kidney | HFB_1_C_22:0_ | 0,0 | 0,0 | 0,0 | ± | 0,00 |  | FB_1_C_22:0_ | 0,0 | 0,0 | 0,0 | ± | 0,00 |
| FB_1_ | 0,5 | Liver | HFB_1_C_22:0_ | 0,0 | 0,0 | 0,0 | ± | 0,00 |  | FB_1_C_22:0_ | 2,9 | 6,4 | 4,7 | ± | 1,71 |
| FB_1_ | 1 | Liver | HFB_1_C_22:0_ | 0,0 | 0,0 | 0,0 | ± | 0,00 |  | FB_1_C_22:0_ | 1,2 | 4,0 | 2,6 | ± | 1,40 |
| FB_1_ | 2 | Liver | HFB_1_C_22:0_ | 0,0 | 0,0 | 0,0 | ± | 0,00 |  | FB_1_C_22:0_ | 2,9 | 4,6 | 3,8 | ± | 0,82 |
| HFB_1_ | 1 | Liver | HFB_1_C_22:0_ | 61,0 | 45,8 | 53,4 | ± | 7,60 |  | FB_1_C_22:0_ | 0,0 | 0,0 | 0,0 | ± | 0,00 |
| control | 0 | Liver | HFB_1_C_22:0_ | 0,0 | 0,0 | 0,0 | ± | 0,00 |  | FB_1_C_22:0_ | 0,0 | 0,0 | 0,0 | ± | 0,00 |

**Table S1** continued

| treatment | | analysis | | | | | | | | | | | | | |
| --- | --- | --- | --- | --- | --- | --- | --- | --- | --- | --- | --- | --- | --- | --- | --- |
| compound | dose mg/kg BW | tissue | analyte | a | b | Mean |  | S.D. |  | analyte | a | b | mean |  | S.D. |
| FB_1_ | 1 | Kidney | HFB_1_C_24:0_ | 0,0 | 0,0 | 0,0 | ± | 0,00 |  | FB_1_C_24:0_ | 3,0 | 2,4 | 2,7 | ± | 0,29 |
| FB_1_ | 2 | Kidney | HFB_1_C_24:0_ | 0,0 | 0,0 | 0,0 | ± | 0,00 |  | FB_1_C_24:0_ | 5,0 | 4,4 | 4,7 | ± | 0,29 |
| HFB_1_ | 1 | Kidney | HFB_1_C_24:0_ | 3,0 | 2,7 | 2,9 | ± | 0,20 |  | FB_1_C_24:0_ | 0,0 | 0,0 | 0,0 | ± | 0,00 |
| control | 0 | Kidney | HFB_1_C_24:0_ | 0,0 | 0,0 | 0,0 | ± | 0,00 |  | FB_1_C_24:0_ | 0,0 | 0,0 | 0,0 | ± | 0,00 |
| FB_1_ | 0,5 | Liver | HFB_1_C_24:0_ | 0,0 | 0,0 | 0,0 | ± | 0,00 |  | FB_1_C_24:0_ | 11,7 | 12,3 | 12,0 | ± | 0,28 |
| FB_1_ | 1 | Liver | HFB_1_C_24:0_ | 0,0 | 0,0 | 0,0 | ± | 0,00 |  | FB_1_C_24:0_ | 2,1 | 7,3 | 4,7 | ± | 2,58 |
| FB_1_ | 2 | Liver | HFB_1_C_24:0_ | 0,0 | 0,0 | 0,0 | ± | 0,00 |  | FB_1_C_24:0_ | 9,0 | 17,6 | 13,3 | ± | 4,29 |
| HFB_1_ | 1 | Liver | HFB_1_C_24:0_ | 25,0 | 23,5 | 24,2 | ± | 0,78 |  | FB_1_C_24:0_ | 0,0 | 0,0 | 0,0 | ± | 0,00 |
| control | 0 | Liver | HFB_1_C_24:0_ | 0,0 | 0,0 | 0,0 | ± | 0,00 |  | FB_1_C_24:0_ | 0,0 | 0,0 | 0,0 | ± | 0,00 |
| FB_1_ | 0,5 | Kidney | HFB_1_C_24:1_ | 0,0 | 0,0 | 0,0 | ± | 0,00 |  | FB_1_C_24:1_ | 3,6 | 3,5 | 3,6 | ± | 0,06 |
| FB_1_ | 1 | Kidney | HFB_1_C_24:1_ | 0,4 | 0,0 | 0,2 | ± | 0,18 |  | FB_1_C_24:1_ | 4,1 | 4,6 | 4,3 | ± | 0,28 |
| FB_1_ | 2 | Kidney | HFB_1_C_24:1_ | 0,1 | 0,0 | 0,0 | ± | 0,04 |  | FB_1_C_24:1_ | 5,7 | 4,2 | 4,9 | ± | 0,73 |
| HFB_1_ | 1 | Kidney | HFB_1_C_24:1_ | 7,9 | 10,8 | 9,3 | ± | 1,43 |  | FB_1_C_24:1_ | 0,0 | 0,0 | 0,0 | ± | 0,00 |
| control | 0 | Kidney | HFB_1_C_24:1_ | 0,0 | 0,0 | 0,0 | ± | 0,00 |  | FB_1_C_24:1_ | 0,0 | 0,0 | 0,0 | ± | 0,00 |
| FB_1_ | 0,5 | Liver | HFB_1_C_24:1_ | 0,0 | 0,0 | 0,0 | ± | 0,00 |  | FB_1_C_24:1_ | 9,2 | 11,8 | 10,5 | ± | 1,28 |
| FB_1_ | 1 | Liver | HFB_1_C_24:1_ | 0,0 | 0,1 | 0,1 | ± | 0,07 |  | FB_1_C_24:1_ | 3,4 | 6,3 | 4,9 | ± | 1,46 |
| FB_1_ | 2 | Liver | HFB_1_C_24:1_ | 0,4 | 0,0 | 0,2 | ± | 0,21 |  | FB_1_C_24:1_ | 9,0 | 11,1 | 10,1 | ± | 1,05 |
| HFB_1_ | 1 | Liver | HFB_1_C_24:1_ | 156,0 | 140,5 | 148,3 | ± | 7,75 |  | FB_1_C_24:1_ | 0,0 | 0,0 | 0,0 | ± | 0,00 |
| control | 0 | Liver | HFB_1_C_24:1_ | 0,2 | 0,1 | 0,2 | ± | 0,07 |  | FB_1_C_24:1_ | 0,0 | 0,0 | 0,0 | ± | 0,00 |
| FB_1_ | 0,5 | Kidney | Total NAHFB_1_ | 0,1 | 0,4 | 0,3 | ± | 0,15 |  | Total NAFB_1_ | 16,7 | 19,9 | 18,3 | ± | 1,60 |
| FB_1_ | 1 | Kidney | Total NAHFB_1_ | 0,7 | 0,3 | 0,5 | ± | 0,20 |  | Total NAFB_1_ | 26,4 | 23,3 | 24,9 | ± | 1,55 |
| FB_1_ | 2 | Kidney | Total NAHFB_1_ | 0,3 | 0,6 | 0,5 | ± | 0,15 |  | Total NAFB_1_ | 27,7 | 27,2 | 27,5 | ± | 0,25 |
| HFB_1_ | 1 | Kidney | Total NAHFB_1_ | 40,7 | 57,3 | 49,0 | ± | 8,30 |  | Total NAFB_1_ | 0,0 | 0,0 | 0,0 | ± | 0,00 |
| control | 0 | Kidney | Total NAHFB_1_ | 0,0 | 0,1 | 0,1 | ± | 0,05 |  | Total NAFB_1_ | 0,0 | 0,0 | 0,0 | ± | 0,00 |
| FB_1_ | 0,5 | Liver | Total NAHFB_1_ | 0,6 | 0,7 | 0,7 | ± | 0,05 |  | Total NAFB_1_ | 33,8 | 37,9 | 35,9 | ± | 2,05 |
| FB_1_ | 1 | Liver | Total NAHFB_1_ | 0,2 | 0,7 | 0,5 | ± | 0,25 |  | Total NAFB_1_ | 12,6 | 27,0 | 19,8 | ± | 7,20 |
| FB_1_ | 2 | Liver | Total NAHFB_1_ | 0,4 | 0,3 | 0,4 | ± | 0,05 |  | Total NAFB_1_ | 32,3 | 43,2 | 37,8 | ± | 5,45 |
| HFB_1_ | 1 | Liver | Total NAHFB_1_ | 287,2 | 269,2 | 278,2 | ± | 9,00 |  | Total NAFB_1_ | 0,0 | 0,0 | 0,0 | ± | 0,00 |
| control | 0 | Liver | Total NAHFB_1_ | 0,0 | 0,0 | 0,0 | ± | 0,00 |  | Total NAFB1 | 0,0 | 0,0 | 0,0 | ± | 0,00 |
